# The meaning of self-acceptance

**DOI:** 10.7554/eLife.93328

**Published:** 2023-10-12

**Authors:** Uyen Vo

**Affiliations:** 1 Department of Molecular & Cell Biology, University of California, Berkeley Berkeley United States

**Keywords:** sparks of change, research culture, equity diversity inclusion, being neurodivergent in academia, neurodiversity, ADHD

## Abstract

A research technician describes how receiving an ADHD diagnosis allowed her to re-examine how she sees herself and her work.

*You need to pay more attention. If you’d rather watch the birds than follow the lesson, then get out of my classroom! You’ve got potential, but you aren’t trying hard enough to reach it. Why can’t you be like other children?*

…

*Why are you this way?*

The relentless chorus grew louder in my head, eventually drowning out all other sounds. I felt trapped by the voices, by the grip of the chair, by a tsunami of pain suddenly crashing onto my chest. In a lecture hall of 300 undergraduate students, nobody seemed to notice I was gasping for air.

I had received so many of these comments growing up. When I relocated from Vietnam to the United States to study cell biology, I thought I might leave them behind. But no matter what I did, the voices kept plaguing my mind.

I had always felt deep down that something was amiss about me – after all, no one else seemed to require exhausting amounts of mental effort to pay attention in class or to navigate mundane tasks – but I had never been able to understand why this was the case. In a way, it was easier to just comply with the deceivingly simple mantra drilled into me since childhood: “You just need to try harder”.

Once, in a moment of desperation during my freshman year at university, I turned to Google. Amongst the long list of possible answers that ranged from sleep deprivation to brain tumors, I stumbled upon attention-deficit/hyperactivity disorder – ADHD. I went through the list of symptoms, ticking them off one after the other. Hope flickered as some semblance of clarity unfolded. Still, most articles painted a profile that felt too reductive, describing restless children uninterested in school and destined to fail in academia. I knew this wasn’t me. I enjoyed my science classes, my grades were good overall and I wasn’t the restless ball of energy people labeled as hyperactive. Maybe I didn’t have ADHD after all. Maybe I was merely moving at a slower pace, maybe I just needed to try harder – there it was again, the familiar whisper of self-doubt. The flicker of hope quickly faded away, leaving me to grapple with uncertainty once more.

My suspicions resurfaced in my last month as an undergraduate when one of my friends and fellow classmates opened up about her diagnosis. Her struggles resonated with mine, yet she didn’t seem to fit the stereotypical portrayal of ADHD either. She encouraged me to get assessed, showing unfailing belief in my potential. The weight of facing my challenges alone lifted and with her by my side, hope ignited again. This time it was here to stay.

I looked for an affordable ADHD assessment as soon as I got a job as a research technician after graduating. Shifting through a seemingly endless list of options, I finally managed to find the only provider in my area who was covered by my work health insurance and therefore wouldn’t cost me thousands of dollars.

*“Your symptoms and test results are consistent with ADHD”*’.

I felt relief, exhilarating relief. Finally, it all made sense. The hurricane of thoughts hurling through my mind from the moment I wake up, stopping me from focusing on the papers I want to read. The dispersed attention that unnecessarily latched onto random bits of a presentation. The unreasonable rage at the noise of the PCR machine, the beep of the lab door code, the squeaking chairs at seminars, the constant tickle of the hair poking my skin. The steps missed when following written protocols. The time blindness. The paralytic agony when a criticism feels like rejection. The unproductive workaholism.

The fraud I thought I was.

The diagnosis brought the clarity that had been missing my whole life, yet I couldn’t help but also feel betrayed. I thought about my childhood, growing up poor in a country where awareness of any neurodevelopmental conditions is a luxury. There is no translation for the word ‘neurodiversity’ in Vietnamese or even an equivalent. Wellbeing, mental health, or neurodivergence are shameful taboos that parents usually dismiss, ironically, by saying it’s all “in your head”. The cure to inattentiveness, forgetfulness, or impulsivity, in their opinion? Pay more attention. Remember better. Control yourself.

I’d tried so hard but these simple “solutions” never worked for me, as if an invisible wall stood there in front of me. At home and school, adults attempted to ‘correct’ what I know now were my ADHD traits by belittling me, punishing me, or beating me. Little by little, I internalized these criticisms. By the time I left for the US, I believed that I was lazy, incompetent, and disobedient. Gullibly, I hoped that such self-condemnation would push me to grow and make me a better scientist. Instead, each day became an uphill battle. It took me too long to realize I was drowning in guilt and shame. The diagnosis finally opened my eyes: I had spent so much time blaming myself for not being able to fix what I didn’t know was there to be fixed.

It took a comment from a postdoc in my lab for me to see what this could mean. Around the time he told me that eLife was interested in hearing from neurodivergent scientists, he encouraged me to think about how I would want my ADHD to be pictured. I understood then that I had the freedom to reshape my story. I could decide to change the internal lens through which I viewed my worth and start being kinder to myself. In that moment, I snapped back from an eternity of crisis. For the first time, I recognized that my ADHD traits could also be assets, the building blocks that make me unique as a scientist. My ability to hyperfocus has allowed me to become fully immersed in refining my technical skills or analyzing data. My thoughts may often get side-tracked, but this has also led to creative questions and new observations.

In a way, this fresh perspective brought challenges of its own. To say that all the burdens of ADHD can be harnessed for good is wishful thinking. I believed I had to create a narrative that would allow me to acknowledge my ADHD without feeling defective, to forgive it while taking accountability for my mistakes, and to recognize its strengths while not romanticizing it as a superpower. I saw self-acceptance as a goal, something I could only reach after discovering this perfect balance and deciding on a final, positive stance about myself and ADHD. As I struggled to reconcile all these thoughts, I thought perhaps external insights could help.

I mustered the courage to share the news of my diagnosis at work. I was blessed to receive the support of my lab members and my PI, who at times believed in me more than I believed in myself. However, not everyone in academia has been as supportive. I’ve received comments about how ADHD isn’t real or that I couldn’t possibly have received a diagnosis given how ‘high-functioning’ I was. I was warned that I had fallen victim to a narrative that “medicalizes normal behaviors”, while also being told I was taking “the easy way out” for seeking medication to manage my ADHD. These dismissive remarks stripped me of my true identity, leaving me feeling vulnerable and invisible. They confirmed my fears that people would think I was using ADHD as a feeble excuse for falling short; they fed into my doubts about the validity of my diagnosis, and whether I could succeed as a scientist in this environment.

I know I share these experiences with many other neurodivergent friends and researchers. There’s an urgent need for safe spaces that offer neurodivergent academics validation and solidarity so we can thrive and contribute our unique perspectives to science. It’s disheartening to see that need ignored so often. Nevertheless, I find solace in knowing that I am not alone in coping with ADHD and in challenging the misconceptions around it. Through openness and advocacy, I am carving a path of acceptance and understanding, not only for myself but also for others who may share similar struggles.

Ultimately, I’ve realized that I don’t need to find a perfect balance for myself. I certainly don’t owe it to anyone to create an ADHD narrative that accommodates everybody’s opinions. I will probably always oscillate between different emotions about my diagnosis; sometimes I may think that I’m bound to struggle forever, and sometimes I may feel empowered by it. And that’s OK. This is what self-acceptance is for me now. It’s not about having a rosy outlook on myself or glossing over the unpleasant emotions arising from my doubts and self-awareness. Instead, it’s embracing and welcoming it all, the good and the bad, the certain and the uncertain, the moments of strength and vulnerability alike.

## About this article

This Sparks of Change article is part of a series of articles on being neurodivergent in academia, which includes a list of tips, resources and tools collated by neurodivergent scientists.

